# Development to the blastocyst stage, the oxidative state, and the quality of early developmental stage of porcine embryos cultured in alteration of glucose concentrations in vitro under different oxygen tensions

**DOI:** 10.1186/1477-7827-4-54

**Published:** 2006-11-06

**Authors:** Ni Wayan Kurniani Karja, Kazuhiro Kikuchi, Mokhamad Fahrudin, Manabu Ozawa, Tamás Somfai, Katsuhiko Ohnuma, Junko Noguchi, Hiroyuki Kaneko, Takashi Nagai

**Affiliations:** 1 Research Support Center, Swine and Poultry Feeding Management Laboratory, National Institute of Livestock and Grassland Science, Tsukuba, Ibaraki 305-0901, Japan; 2 Department of Animal Sciences, Reproductive Biology Research Unit, National Institute of Agrobiological Sciences, Tsukuba, Ibaraki 305-8602, Japan; 3 Department of Animal Reproduction, Faculty of Veterinary Medicine, Gadjah Mada University, Yogyakarta 55281, Indonesia; 4 Faculty of Veterinary Medicine, Bogor Agricultural University, Bogor 16680, Indonesia

## Abstract

**Background:**

Recent work has shown that glucose may induce cell injury through the action of free radicals generated by autooxidation or through hypoxanthine phosphoribosyltransferase inhibition. The effect of glucose during early in vitro culture (IVC) period of porcine embryos on their developmental competence, contents of reactive oxygen species (ROS) and glutathione (GSH), and the quality of the blastocysts yielded was examined.

**Methods:**

In vitro matured and fertilized porcine oocytes were cultured for the first 2 days (Day 0 = day of fertilization) of IVC in NCSU-37 added with 1.5 to 20 mM glucose (Gluc-1.5 to -20 groups) or pyruvate and lactate (Pyr-Lac group). The embryos in all groups were cultured subsequently until Day 6 in NCSU-37 with 5.5 mM added glucose. The ROS and GSH level were measured at Day 1 and 2. DNA-fragmented nuclei and the total cell numbers in blastocyst were evaluated by TUNEL-staining at Day 6.

**Results:**

Under 5% oxygen the blastocyst rates and total cell numbers in the blastocysts in all glucose groups were significantly lower than that in the Pyr-Lac group. Similar result in blastocyst rate was found under 20% oxygen (excluding the Gluc-10 group), but total cell numbers in the blastocysts was similar among the groups. At both oxygen tensions, the H2O2 levels of Day 1 embryos in all glucose groups were significantly higher than that in the Pyr-Lac group, while only the Gluc-1.5 group of Day 2 embryos showed a significantly higher H2O2 level than that in the Pyr-Lac group. The GSH contents of either Day 1 or Day 2 embryos developed under 5% oxygen were similar among the groups. Only the content of Day 2 embryos in 1.5 mM group was significantly lower than the embryos in the Pyr-Lac group under 20% oxygen. Total cell numbers in the blastocysts (except in the Gluc-20 group) were significantly lower in the embryos cultured under 20% oxygen than 5% oxygen. Only the Gluc-20 blastocysts developed under 5% oxygen showed significantly higher DNA fragmentation rate than those of Pyr-Lac blastocysts.

**Conclusion:**

These results show that a decrease in developmental ability of embryos cultured by use of glucose instead of pyruvate and lactate after the ferilization may be due to the rise in ROS generation in Day 1 embryos. Moreover, results from this study suggest that the concentration of glucose in the medium that can be used by the Day 1–2 embryos is limited to 3.5 mM and exposure to higher glucose concentrations does not improve embryo development.

## Background

Glucose is the main fuel for most cells, and its importance as an energy substrate has led to intense research on its cellular metabolites and the mechanism controlling its uptake. A vast number of studies have been conducted on glucose metabolism in mammalian preimplantation embryos. Most of the reports have shown that the presence of glucose in early preimplantation embryos up to the 8-cell stage is detrimental to further embryo development in vitro in several species, including the hamster [1], mouse [2], rat [3], cow [4], sheep [5], and human [6]. On the other hand, nutrient uptake studies carried out on porcine embryos have shown that the embryos consume glucose and produce lactate at all stages of development. These studies have also shown that glucose-utilizing pathways are active throughout embryonic development, and that glucose does contribute as an energy source [7-11]. Therefore, glucose-containing media are commonly used for producing porcine embryos in vitro [9,12,13]. However, it also remains unclear whether the metabolic profiles of embryos cultured in glucose-containing medium are more or less reflective of correct in vivo porcine embryo metabolism, since the concentration of glucose commonly used in the culture medium is much higher than that in the oviduct, where in vivo porcine embryos are exposed to 0.17 mM glucose [14]. Moreover, it has been also postulated that metabolic activity of pig embryos, which is reflected by glucose and pyruvate uptake as well as lactate production, differs depending on the culture medium used [9].

Certain types of cells are known to suffer from glucose toxicity. It has been suggested that glucose may induce cell injury through the action of free radicals generated by autooxidation or through hypoxanthine phosphoribosyltransferase (HPRT) inhibition, which leads to the conversion of hypoxanthine into xanthine with production of reactive oxygen species (ROS) [15-17]. Reactive oxygen species are highly reactive with complex cellular molecules such as proteins, lipids, and DNA, and cause serious dysfunction such as enzyme inactivation [18], mitochondrial abnormality [19], or DNA fragmentation [17]. High concentrations of ROS in the microenvironment surrounding the preimplantation embryo in vitro may disturb the balance between the formation of ROS and antioxidants, leading to oxidative stress, which is generally thought to be harmful for embryonic development [17,20-22].

Our objectives were to examine the effects of supplementation with various glucose concentrations as a sole energy substrate during the first 2 days of culture (early development stage) on 1) the development ability of embryos to the blastocyst stage, 2) reactive oxygen species production, as measured by the relative concentration of hydrogen peroxide (H_2_O_2_), 3) glutathione (GSH) content, and 4) blastocyst quality (as defined by the total number of cells and incidence of DNA fragmentation in the blastocyst).

## Materials and methods

### Procedures for in vitro maturation (IVM) and in vitro fertilization (IVF) of porcine oocytes

The procedures for oocyte collection, IVM, and IVF have been described previously [23,24]. Presumptive zygotes were obtained after 3 h of IVF and cumulus cells were removed by pipetting vigorously. They were subsequently cultured randomly in groups of 50–55 embryos per 500 μl of the media to which they had been assigned, as described below.

### Experimental design

The basic medium used for embryo culture was glucose-free NCSU-37 [12] containing 4 mg/ml BSA and 50 μM β-mercaptoethanol (IVC medium). The basic medium was then modified by supplementation with sodium pyruvate and sodium lactate or various concentrations of glucose. Generally, culture was performed at 38.5°C and 5% CO_2 _under 5% O_2 _and 90% N_2_. To test the effects of different atmospheric conditions, embryos were also cultured under 5% CO_2 _in air (which contains approximately 20% O_2_).

### Experiment 1: Effect of glucose concentration on development of IVP porcine embryos

To determine the effect of increasing glucose concentration as a sole energy substrate during early embryonic development on the developmental competence of porcine embryos to the blastocyst stage in vitro, presumptive zygotes were cultured from Days 0 to 2 (the day of IVF was defined as Day 0) in IVC medium containing 1.5, 3.5, 5.5, 10, or 20 mM glucose (Gluc-1.5, Gluc-3.5, Gluc-5.5, Gluc-10, and Gluc-20 groups, respectively). Zygotes were also cultured in IVC medium containing 0.17 mM pyruvate and 2.73 mM lactate (Pyr-Lac group), the standard medium used in our culture system [23]. Subsequently, embryos in all energy supplement groups were cultured until Day 6 in IVC medium supplemented with 5.5 mM glucose.

### Experiment 2: Measurement of intracellular H_2_O_2 _levels

To determine the quantity of H_2_O_2 _produced by the embryos that had been cultured in the presence or absence of glucose during the first 2 days of IVC, the relative intensity of H_2_O_2 _production in Day 1 (at the one-cell stage; after 20–22 h of IVC) or Day 2 (at the 4-cell stage; after 44–48 h of IVC) embryos in each energy supplement group was measured using 2',7' -dichlorodihydrofluorescein diacetate (DCHFDA; Sigma) [25,26]. DCHFDA is membrane permeant and, therefore, is able to diffuse readily into cells. Within the cell, acetate groups are hydrolyzed by intracellular esterase, forming 2',7'-dichlorodihydrofluorescein (DCHF). DCHF then fluoresces when it is oxidized by H_2_O_2 _to 2',7'-dichlorofluorescein diacetate (DCF). The level of DCF produced within the cells is related to the concentration of peroxide present; thus, its fluorescent emission enables measurement of the cellular peroxide level [25,26]. Embryos in each energy supplement group were incubated for 15 min in IVC medium containing 10 μM DCHFDA, and then washed in fresh IVC medium before being placed on a glass slide and covered with a cover slip. The fluorescence emissions were recorded as JPEG files using a digital camera (DP 12; BX 51; Olympus, Tokyo, Japan) attached to a fluorescent microscope (BX 51; Olympus) after excitation at 480 nm and emission at 510 nm. The recorded fluorescent image was converted to TIFF files by using Adobe Photoshop 7.0 (Adobe Systems Inc., San Jose, CA), then analyzed using Scion Image Beta 4.02 (Scion Co., Maryland, USA). The fluorescent image of the embryos was measured by counting the number of pixels after inversion of the color.

### Experiment 3: Measurement of intracellular GSH content

Intracellular GSH content was measured as described by previous studies [27,28]. For each replicate, we placed pools of 10–15 Day 1 embryos or 20–25 Day 2 embryos in 5 μl of 0.2 M sodium phosphate buffer containing 10 mM Na_2_-EDTA (pH 7.2) and 5 μl of 1.25 M phosphoric acid in microtubes and then all the embryos were frozen at -80°C. The concentrations of GSH in the embryos were determined by dithionitrobenzoic acid – glutathione disulphide (DTNB-GSSG) reductase recycling assay [29]. Briefly, the samples were thawed, and then 175 μl sodium phosphate buffer containing 0.33 mg/ml nicotinamide adenine dinucleotide phosphate (NADPH) (Sigma), 25 μl of 6 mM DTNB (Wako Pure Chemical Industries, Ltd., Osaka, Japan), and 40 μl of water were added to each sample tube. The samples were warmed at room temperature for 15 min, and then the assay was initiated with the addition of 5 μl of 125 IU glutathione disulphide reductase (Wako). Absorbance was measured five times by spectrophotometer (DU7500; Beckman Coulter, Inc., CA, USA) at 30-s intervals at a wavelength of 412 nm. A GSH standard and a sample blank lacking GSH was also assayed. Standards were prepared for each assay, and the total GSH content per sample was determined from a standard curve of GSH. The GSH concentration per embryo was calculated by dividing the total concentration per sample by the number of embryos present in the sample.

### Experiment 4: TUNEL assay

DNA fragmentation of blastocyst was analyzed by using a combined technique for simultaneous nuclear staining and TUNEL (in situ cell death detection system; Roche Diagnostic Corporation, Indianapolis, IN, USA) by a modification of the procedures previously described by Karja et al. [30]. Blastocysts on Day 6 in each group were fixed overnight at 4°C in 3.7% (w/v) paraformaldehyde diluted in phosphate buffer saline (PBS). After overnight fixation, embryos were washed thrice in PBS containing 3% (w/v) polyvinyl alcohol (PVA) and permeabilized in 0.5% (v/v) Triton X-100 for 60 min, and then incubated in a blocking solution (PBS containing 10 mg/ml BSA) overnight at 4°C. As positive controls, one or two embryos per TUNEL analysis were incubated in 1000 U/ml deoxyribonuclease I (DNase I; Sigma) for 20 min. After washing in PBS-PVA, the positive controls and all experimental embryos were incubated in TUNEL reaction cocktail at 37°C for 1 h in the dark. The embryos were then counterstained with 50 μg/ml propidium iodide (PI) for 20 min to label all nuclei. The embryos were washed extensively and mounted with slight coverslip compression in an anti-bleaching solution (Slow-Fade; Molecular Probes, Eugene, OR, USA). The embryos were examined under a confocal laser-scanning microscope (IX-71, Olympus) fitted with 25/40 × PL Fluotar/0.75 oil objectives, and an argon/krypton laser was used for excitation at wavelengths of 488 and 568 nm for detection of the TUNEL reaction and PI, respectively. A complete Z series of 20–27 optical sections at 3- to 4-μm intervals was acquired from each embryo. The images were reconstructed using Fluoview software (Olympus). Each optical section of the blastocyst was analyzed for total number of nuclei and number of TUNEL-labeled nuclei. In the present study, the ratio of number of TUNEL-labeled nuclei to total number cells was defined as DNA fragmented nucleus index.

### Statistical analysis

Data were expressed as means ± SEM. The percentages of embryos that developed to the blastocyst stage and the DNA-fragmented nucleus index were subjected to arcsine transformation before analysis. Then all data, including the arcsine-transformed percentages of replications of the rate of blastocyst formation and the DNA-fragmented nucleus index; the data on intracellular content of GSH and intracellular level of H_2_O_2 _and the data on mean number of cells in the blastocyst, were subjected to ANOVA by using the General Linear Models procedures of the Statistical Analysis System (SAS Institute Inc., Cary, NC) and were then analyzed by the Duncan multiple range test.

## Results

### Experiment 1: Rates of development to the blastocyst stage

**Figure 1:**
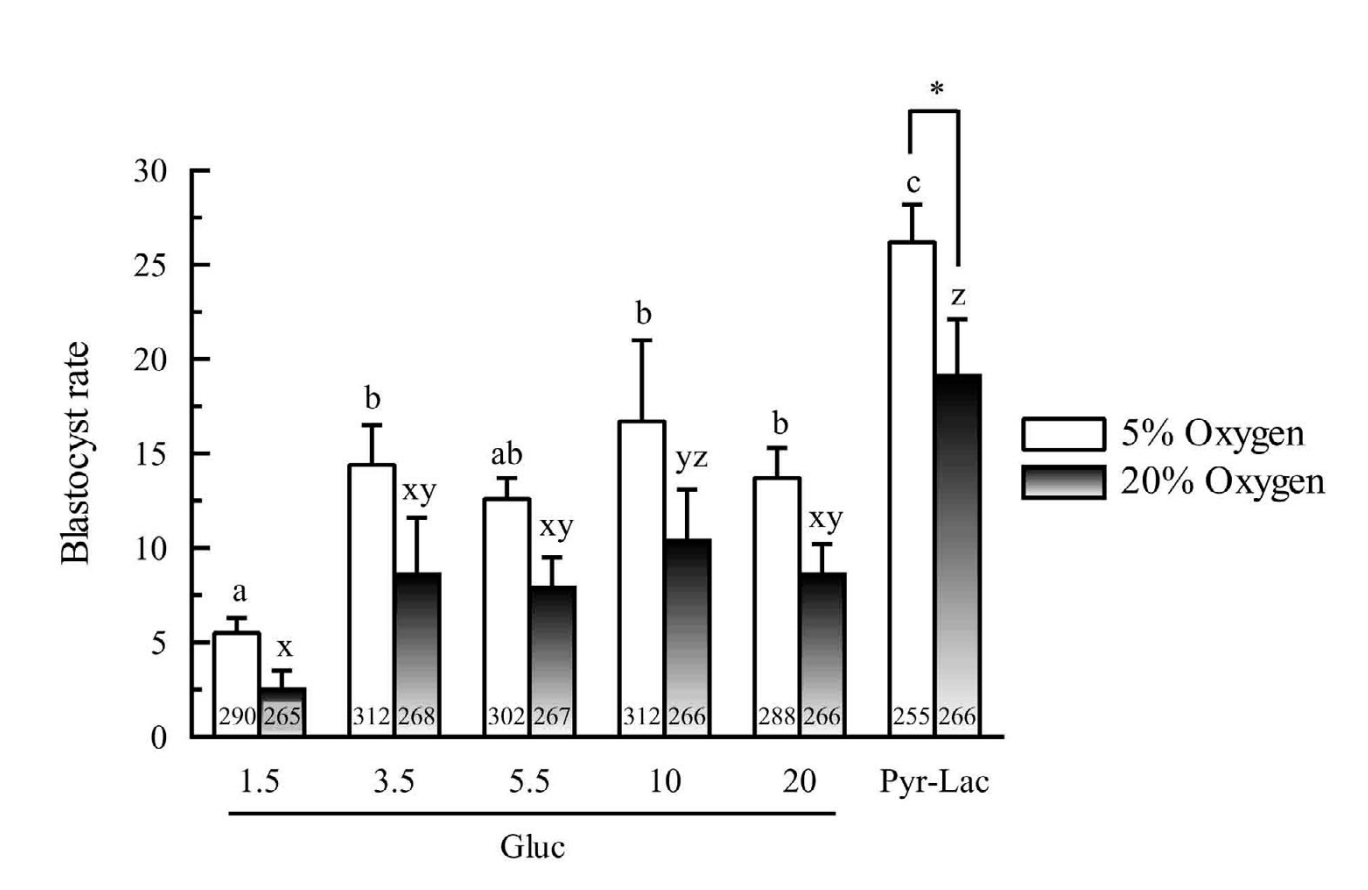
Developmental rates to the blastocyst stage of in vitro-produced porcine embryos developed under either 5% or 20% oxygen tensions: comparison of different energy substrate supplements during the first 2 days of IVC. Blastocyst rates are expressed based on the number of examined embryos. Six replicate trials were carried out. Within each end point, bars with different letters (a-c and x-z) are significantly different for 5% and 20% oxygen tension treatments, respectively (*P *< 0.01). There was no statistical difference within each energy substrate supplement group across oxygen tension treatments, with the exception of the Pyr-Lac groups (*P *< 0.01). The numbers on the bars represent the number of oocytes fertilized, cultured and analyzed.

In vitro development of porcine zygotes to the blastocyst stage in IVC medium with increased glucose concentration or in the medium supplemented with pyruvate and lactate during the first 2 days of IVC, under two different oxygen tensions (5% or 20%), are shown in figure 1. Of the embryos cultured under 5% oxygen, the proportions of the embryos that developed to the blastocyst stage were significantly higher in the Pyr-Lac group than those in the glucose groups at any concentration. Similarly, under 20% oxygen tension, the rates of blastocyst formation in 4 of the glucose groups, except for the Gluc-10 group, were significantly lower than that in the Pyr-Lac group. There was no significant difference in the rates of blastocyst formation within each energy supplement group across oxygen tension treatments, except for the Pyr-Lac group (*P *< 0.01).

### Experiment 2: Generation of intracellular H_2_O_2_

**Figure 2:**
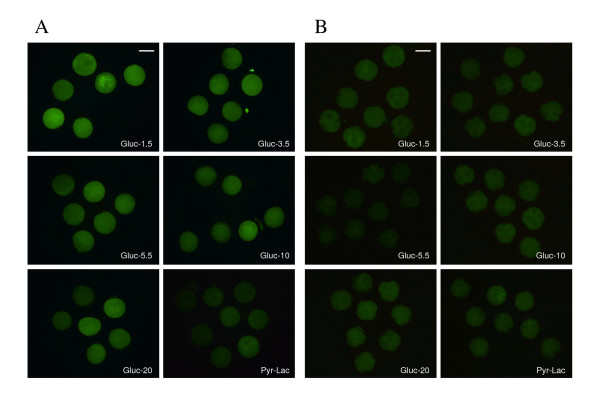
Fluorescent photomicrographs with 2',7' -dichlorodihydrofluorescein diacetate of porcine embryos on Day 1 at the one-cell stage (A) and embryos on Day 2 at the 4-cell stage (B). Embryos were cultured in IVC medium containing 1.5 to 20 mM glucose or pyruvate-lactate. Scale bars represent 100 μm.

**Figure 3:**
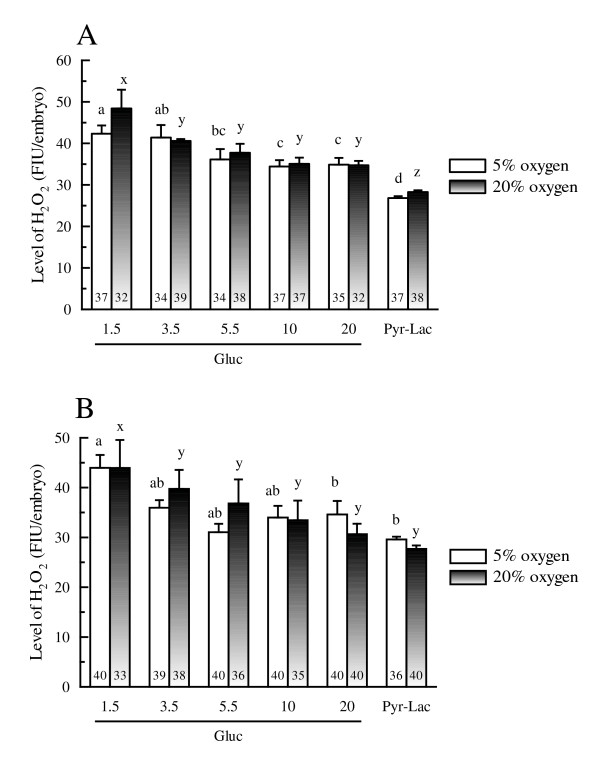
Hydrogen peroxide levels in Day 1 (A) and Day 2 (B) porcine embryos cultured either in IVC medium containing 1.5 to 20 mM glucose or pyruvate-lactate. Values represent arbitrary fluorescence intensity units (FIU). Five replicate trials were carried out. Within each end point, bars with different letters (a-d and x-z) are significantly different for 5% and 20% oxygen tension treatments, respectively (*P *< 0.01). No significant differences were found in H_2_O_2 _levels within each energy substrate supplement group across oxygen tension treatments. The numbers on the bars represent the number of embryos used for the assay.

Fluorescent photomicrographs of Day 1 and 2 embryos stained with DCHFDA are shown in figure 2A and 2B, respectively. Intracellular H_2_O_2 _levels of Day 1 and 2 embryos after analysis by Scion Image Beta 4.02 are shown in figure 3A and 3B, respectively. At both oxygen tensions, the H_2_O_2 _level of Day 1 embryos at any glucose concentration was significantly higher than that in embryos cultured in Pyr-Lac (*P *< 0.01). Moreover, H_2_O_2 _level in the Gluc-1.5 group was significantly higher than those in the other glucose groups (*P *< 0.05). In Day 2 embryos, the level significantly differed only in the embryos of the 1.5 mM group compared with the embryos in the Pyr-Lac group under both oxygen tensions (*P *< 0.05). No significant differences in H_2_O_2 _levels of Day 1 or Day 2 embryos were found within each energy substrate supplement group across oxygen tension treatments.

### Experiment 3: GSH contents

**Figure 4:**
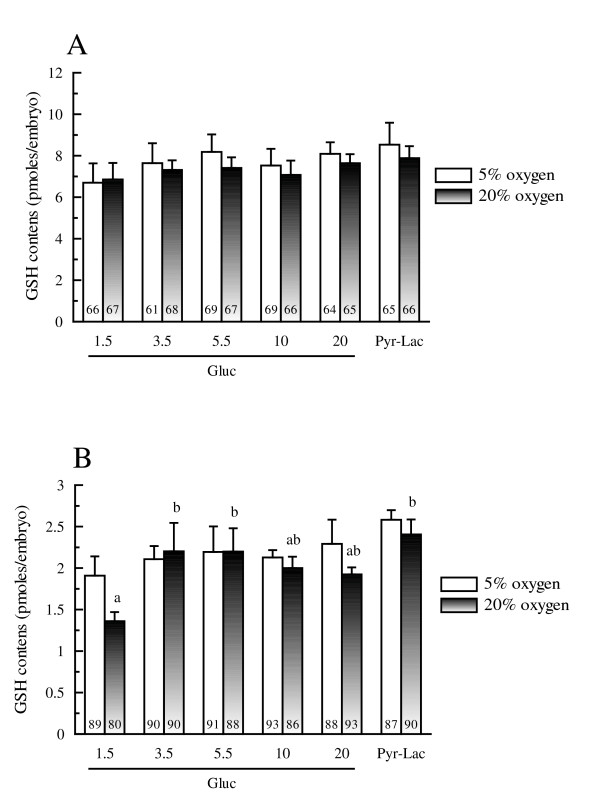
Intracellular GSH contents of Day 1 (A; five replicate trials were carried out) and Day 2 embryos (B; four replicate trials were carried out) cultured in either IVC medium containing 1.5 to 20 mM glucose or pyruvate-lactate. Within each end point, bars with different letters (a, b) are significantly different for 20% oxygen tension treatment (*P *< 0.05). No significant differences were found in GSH level within each energy substrate supplement group across oxygen tension treatments. The numbers on the bars represent the number of embryos used for the assay.

Among the energy substrate supplement groups, no significant differences were found in the GSH contents of Day 1 embryos under either oxygen tension (see figure 4A). Also the GSH content in Day 2 embryos did not differ among energy substrate supplement groups under 5% oxygen tension. However, under 20% oxygen tension, only the Day 2 embryos in the 1.5 mM group had a significantly lower GSH content (*P *< 0.05) than the embryos in the Pyr-Lac group (see figure 4B). Within each energy substrate supplement group, no significant differences were found across oxygen tension treatments in terms of the GSH content of either Day 1 or Day 2 embryos. No interaction was found by ANOVA between the oxygen concentration and the energy supplement groups in intracellular H_2_O_2 _production and GSH levels.

### Experiment 4: Number of cells and DNA fragmentation

**Figure 5:**
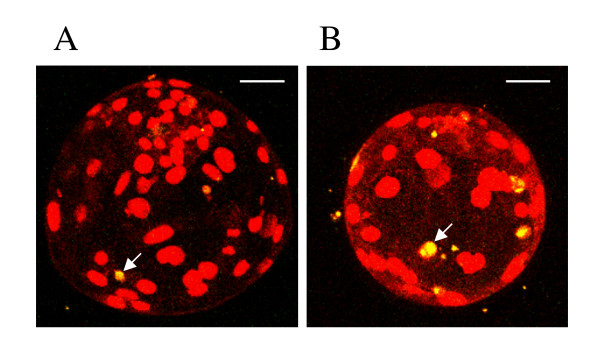
Fragmented TUNEL-labeled nuclei (arrow) were present in almost all blastocysts, irrespective of the culture medium. The blastocysts shown were derived from the Pyr-Lac group (A) or the Gluc-20 group (B) and developed under 5% oxygen. Scale bars represent 20 μm.

**Figure 6:**
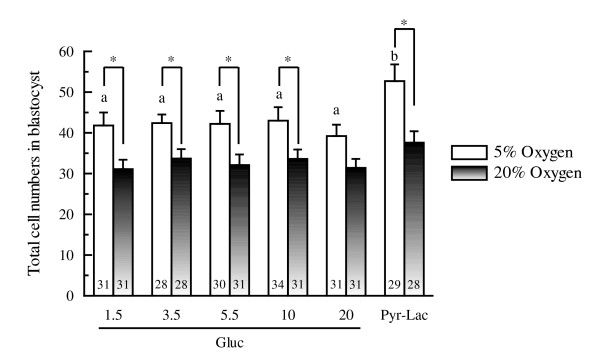
Total number of cells of Day 6 porcine blastocysts developed either under 5% or 20% oxygen tensions: comparison of different culture media during the first 2 days of IVC. Within each end point, bars with different letters (a, b) are significantly different for 5% oxygen tension treatment (*P *< 0.01). There were significant differences (*P *< 0.01) within each energy substrate supplement group (excepting the Gluc-20 group) across oxygen tension treatments. The numbers on the bars represent the number of embryos used for the assay.

**Figure 7:**
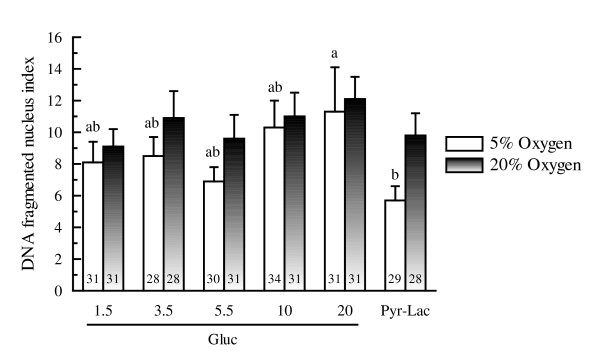
Nuclear DNA fragmentation indices of Day 6 porcine blastocysts developed under either 5% or 20% oxygen tensions: comparison of different culture media during the first 2 days of IVC. Within each end point, bars with different letters (a, b) are significantly different for 5% oxygen tension treatment (*P *< 0.05). There were no significant differences within each energy substrate supplement group across oxygen tension treatments. The numbers on the bars represent the number of embryos used for the assay.

When the number of DNA-fragmented nuclei and the total number of cells per blastocyst were evaluated by TUNEL-staining, DNA-fragmented nuclei were detected in almost all blastocysts, irrespective of the culture conditions (see figure 5). Of the embryos cultured under 5% oxygen tension, the total number of cells in the blastocysts of the Pyr-Lac group was significantly higher (*P *< 0.01) than that in the blastocysts supplemented with glucose at any concentration (see figure 6). In contrast, when cultured under 20% oxygen tension, no significant differences in cell numbers of the blastocysts were found among the energy substrate supplement groups. The cell numbers within each energy supplement group (except for the Gluc-20 group) were significantly higher (*P *< 0.05) under 5% oxygen than under 20% oxygen treatment. Only 20 mM glucose group showed a significantly higher (*P *< 0.05) DNA fragmentation rate than that in the blastocysts of the Pyr-Lac group, however, the fragmentation indices did not differ significantly among the glucose groups (see figure 7). Of the embryos cultured under 20% oxygen, no significant differences in fragmentation rates were found among energy substrate supplement groups. There were no significant differences within each energy supplement group in terms of DNA fragmentation rates across oxygen tension treatments.

## Discussion

Our results demonstrate that addition of glucose at any concentration during the first 2 days of IVC permitted some of the embryos to develop to the blastocyst stage under both oxygen tensions. However, the provision of only glucose as an energy substrate in IVC media failed to give rates of blastocyst development comparable to that achieved in the pyruvate-lactate-containing medium. Culturing of porcine embryos at early developmental stages with pyruvate and lactate in the present study also resulted in an increase of total number of cells in blastocysts developed under 5% oxygen tension. Taken together, these results indicate that pyruvate and lactate appear to be important energy substrates for both early embryonic development in vitro and improvement in the number of cells in the blastocyst. Our observation confirms the previous reports, which reported that pyruvate and lactate seem to be predominant energy substrates for the first cleavage division for porcine embryos [23,31]. However, there were evidences that concentration of pyruvate and lactate when both were present in the medium could affect embryo development [32,33], thus the correct ratio of pyruvate to lactate should be noted. Because the ratio is important for maintenance of the intracellular NAD^+^:NADH ratio and redox equilibrium, thus regulating the oxidation-reduction equilibrium between the cytoplasm and mitocondria [32,34]. In this study, culturing of early porcine embryos in medium with 0.17 mM pyruvate and 2.73 mM lactate for the first 2 days of culture may provide the embryos with more suitable conditions for cellular oxidation reduction equilibrium, resulting in viable embryo growth and development.

In this study, we found that the developmental ability of embryos exposed to 1.5 mM glucose was lower than those of embryos in the other glucose groups. In contrast, when the concentration of glucose was increased to 3.5 mM, a significant increase in blastocyst formation was observed under 5% oxygen tension, and a slight increase was observed under 20% oxygen tension. The rate, however, did not differ from that of Gluc-5.5 group. These results indicate that the efforts to improve blastocyst formation by culturing the embryos in media with glucose at concentrations lower than 5.5 mM was found to be ineffective. At a concentration of 1.5 mM glucose, the necessary energy substrates may not have been present in sufficient concentrations for development of the embryos, resulting in the failure of the embryos to develop to the blastocyst stage. In addition, we also found that the developmental rates of embryos in the Gluc-10 and -20 groups, regardless of the oxygen tension, did not differ from those in other glucose groups, except for Gluc-1.5 group. These results suggest that the concentration of glucose in the medium that can be used by the Day 1–2 embryos is limited to 3.5 mM and exposure to higher glucose concentrations does not improve embryo development. The lack of response to the high glucose concentration in this study could be explained as the limited ability of early porcine embryos to utilize glucose [11] or the embryos use only a fraction of the glucose that they take up [10]. It is known that metabolic activity and substrates preferences of embryos appear to change between early and late cleavages with elevated glucose and oxygen consumption as they approach cavitation [35]. It has been reported that the optimal concentration of glucose varies according to the stage of development [36,37]. In pigs, the first marked increase in glucose metabolism occurs at or about the time of activation of the embryonic genome [38,15]. Therefore the susceptibility of the embryos exposed to the high glucose appears to be depended on the developmental stage.

In this study, we found that H_2_O_2 _level of Day 1 embryos in the Pyr-Lac group embryos at either oxygen tension was lower than that at any of the glucose concentrations tested. Generation of ROS induced by glucose utilization was assumed to be caused by the activation of NADPH oxidase, an enzyme that catalyzes the oxidation of NADPH, generates NADP that serves as a coenzyme of the oxidative arm of the pentose phosphate pathway (PPP) [4,39-42]. Production of superoxide anion and H_2_O_2 _via NADPH oxidase has been described on a rabbit blastocyst surface [43], and the incubation of mouse embryos with an inhibitor of NADPH oxidase induces a dose-dependent reduction in H_2_O_2 _production [26]. Since activity of PPP was higher at zygotes and embryos at the 2-cell stage compared with later developmental stages [9,11], thus enhanced ROS production in embryos cultured with glucose in this study may be associated with the glucose utilization by the early developmental stage of porcine embryos through the pentose phosphate pathway (PPP). Taken together, results from the present study indicate that at least a part of glucose toxicity may be caused by the formation of ROS in Day 1 embryos via glucose metabolism through PPP, resulting in a reduction of both cleavage (data not shown) and blastocyst formation rates. On the other hand, NADPH generated as a result of the PPP activity is also reported to act for the reduction of intracellular GSH, a tripeptide thiol compound that plays a major role in regulating ROS concentrations within cytoplasm [17]. Therefore, we measured the GSH content of early embryos after culturing them with glucose. However, we did not find any effects of glucose on GSH content in both Day 1 and Day 2 embryos. Moreover, we observed a decrease in GSH content of Day 1–2 embryos in all experimental groups. Although the reason was not clear at present, the most likely reason is that it may be due to the inability of early porcine embryos to synthesize GSH. This hypothesis was consistent with a previous work on mouse embryos, which found that preimplantation embryos could not synthesize GSH de novo until the morula or blastocyst stage [44]. During early development, the embryos seem to be dependent on the stored GSH that may be packaged during oocytes development in ovary [38]. Gardiner and Reed [45] observed that the GSH content of mouse embryos decreases continuously from unfertilized oocytes to the blastocyst stage. Thus, it is likely that the observed decrease in GSH content could be due to a depletion of GSH stored in the oocytes combined with an inability of the embryo to synthesize GSH [44,45].

It has been reported that glucose induced cell death through a free radicals-mediated mechanism [16]. Other study demonstrated that the hyperglycemia condition, through the generation of ROS, might lead to the production of ceramide and activation of apoptosis [46]. An exposure of bovine embryos to high concentrations of glucose (10–30 mM) during development from the one-cell to the blastocyst stage resulted in a decrease in the total number of cells in the blastocysts and an increase in the frequency of apoptotic cells [47]. Wongsrikeo et al. [48] reported that replacement of glucose with fructose enhanced embryo quality by increasing the total cell number of blastocysts and decreasing the index DNA fragmented nucleus in blastocysts. In this study, a reduced total cell numbers in blastocysts were observed when the embryos were cultured with glucose for the first 2 days of culture at any concentration as compared with that in the Pyr-Lac group. However, we were unable to detect a significant increase in the number of DNA-fragmented nuclei in blastocysts cultured with increased concentrations of glucose (except for Gluc-20 group that developed under 5% oxygen tension). It has been reported that the response of the cell against oxidative stress can differ widely depending on the intensity of the stress and its duration, and this response varies from the stimulation of cell proliferation to cell arrest or to cell death by apoptosis or necrosis [49]. Therefore, our results suggested that increasing glucose-induced ROS in Day 1 embryos cultured with glucose may have contributed only to cell cycle arrest, as evidenced by reduction of the cleavage rate and blastocyst formation.

The oxygen environment could influence the metabolism [50,51] and the oxidation-reduction potential [32,52] in the embryo. Decreasing the oxygen concentration during culture in vitro has been reported to be beneficial for mammalian embryo development [53-55]. Our results show that, except for the Gluc-20 group, the total number of cells in blastocysts developed under 20% oxygen tension with any energy substrate was lower than that in blastocysts developed under 5% oxygen tension. Although no interaction was found between the oxygen concentration and the energy supplement groups (except for the Pyr-Lac group), blastocyst formation rates of embryos developed under 5% oxygen tension were slightly higher than those in embryos cultured under 20% oxygen tension. These results were consistent with our previous findings [30] and those of other recent reports [53-55] that indicate the beneficial effects of a low oxygen tension during culture in vitro in improving embryonic development and embryo quality. In addition, as mentioned above, since the oxygen effect was suggested is due to an alteration in oxidation-reduction potential in early developmental stages of embryos; at which their development is most dependent on the NAD^+^:NADH ratio as well as pyruvate:lactate ratio [32,52], thus the positive effect of decreasing the oxygen concentration in this study was mostly seen when the embryo was cultured with pyruvate-lactate.

## Conclusion

An exposure of porcine embryos during the first 2 days of IVC to the increased glucose concentrations increased H_2_O_2 _production in Day 1 embryos and impaired the ability of the porcine zygote to cleave and develop to the blastocyst stage. Moreover, results from the this study suggest that the concentration of glucose in the medium that can be used by the day 1–2 emrbyos is limited to 3.5 mM and exposure to higher glucose concentrations does not improve embryo development. In addition, culturing the embryos under 5% oxygen tension improved embryonic development and embryo quality, especially when applied to early developmental stages cultured in pyruvate-lactate-containing medium.
